# Association between in-hospital exclusive breastfeeding and subsequent exclusive breastfeeding until 6 months postpartum in Japan: A cross-sectional study

**DOI:** 10.1371/journal.pone.0310967

**Published:** 2024-10-10

**Authors:** Tomoka Takano, Sumiyo Okawa, Keiko Nanishi, Azusa Iwamoto, Hiromi Obara, Hiroko Baba, Kaori Seino, Yuki Amano, Masahiko Hachiya, Takahiro Tabuchi

**Affiliations:** 1 Bureau of International Health Cooperation, National Center for Global Health and Medicine, Shinjuku-ku, Tokyo, Japan; 2 Institute for Global Health Policy Research, Bureau of International Health Cooperation, National Center for Global Health and Medicine, Shinjuku-ku, Tokyo, Japan; 3 Office of International Academic Affairs, Graduate School of Medicine, The University of Tokyo, Bunkyo-ku, Tokyo, Japan; 4 Division of Epidemiology, School of Public Health, Tohoku University Graduate School of Medicine, Sendai, Miyagi, Japan; University of Oulu: Oulun Yliopisto, FINLAND

## Abstract

Breastfeeding practices during hospitalisation may influence subsequent breastfeeding practices; however, this association has not been well studied in Japan. Therefore, we aimed to examine the association between exclusive breastfeeding (EBF) during hospitalisation and that under 6 months and describe the change in breastfeeding patterns from the first to the sixth month based on the breastfeeding status during hospitalisation. This nationwide cross-sectional internet survey conducted in Japan included 1,433 postpartum women of < 6 months who underwent live singleton deliveries between January 2021 and August 2021. Multivariate Poisson regression was used to analyse the association of first day EBF (24 h after birth) and the first 7 d of EBF, a proxy for the hospitalisation period, with a 24-h recall of EBF before the survey. We described the proportion of breastfeeding practices per age group under 6 months. The rates of EBF during the first day and the first 7 d were 29.7% and 10.0%, respectively. The EBF during the first-day group and the first 7-d group showed significantly higher prevalence ratios of 24-h recall EBF under 6 months of age than the non-EBF groups. The area graphs showed that the rate of EBF was the lowest in the first month of age and gradually increased over time until weaning was initiated. This rate was higher among infants exclusively breastfed during the first 7 d than among those exclusively breastfed on the first day. In conclusion, EBF during hospitalisation was significantly associated with subsequent EBF practice for < 6 months. However, 90% of the infants were supplemented with milk rather than breast milk during hospitalisation. Strengthening breastfeeding support during hospitalisation and after discharge may increase the rate of EBF in children under 6 months, and more mothers and their infants will benefit from breastfeeding.

## Introduction

Breast milk is an essential, free, and readily available nutrient for infants with various benefits [1]. For example, exclusive breastfeeding (EBF) for the first 6 months prevents infection-related infant mortality and morbidity [2, 3], and also, EBF and partial breastfeeding reduces the risk of childhood obesity, and being overweight [4, 5]. Similarly, for maternal health, EBF and partial breastfeeding can reduce the risk of postpartum depression [6, 7], breast cancer [3], ovarian cancer, and type 2 diabetes [8, 9].

Based on these multiple benefits, EBF for the first 6 months of life is recommended by the World Health Organisation (WHO) and the United Nations Children’s Fund (UNICEF) [1]. The global target for EBF is to achieve EBF in at least 50% of children in their first 6 months of life by 2025 [10]. The latest statistics show that 48% of children aged < 6 months were exclusively breastfed globally in 2023, a 10% point increase from 2013 [11]. A study of 113 countries revealed that the EBF rate of < 6 months increased in low- and middle-income countries (LMICs) from 2000 to 2019. Its rate was higher in low-income countries (51.2%) than that in middle-income countries, whereas those in high-income countries (HICs) were not reported due to unavailable data [12]. Another study reported in 2021 that the EBF rate of < 6 months was 18% (0.1–57%) in 30 HICs; however, EBF rates in the remaining 21 HICs covered by this study were not estimated due to unavailable data [13].

The Japanese breastfeeding situation is unique because national policies and clinical practices may not follow global recommendations. For instance, the Breastfeeding and Weaning Support Guide issued by the Japanese government emphasises that healthcare professionals should offer appropriate support, including the use of formula milk when necessary, rather than relying solely on breast milk [14]. The guide does not provide clear guidance on EBF during hospitalisation after delivery [14]. The guide also recommends initiating weaning between 5 and 6 months of age, which is earlier than the WHO recommendations [1, 14]. Only 66 of 1,945 (3.4%) obstetric hospitals and clinics in Japan were accredited as Baby Friendly Hospitals (BFHs) that promote EBF in 2021, implying that breastfeeding support in obstetric hospitals and clinics may vary [15–17].

Furthermore, women are hospitalised for an average of 5 d (for singleton vaginal delivery) after delivery in Japan [18, 19]. This is longer than those in Organisation for Economic Co-operation and Development countries (average 1.4–4.4 d) or LMICs (0.5–6.2 d) [20, 21]. Long periods of hospitalisation may increase the opportunity for women to receive tailored breastfeeding support from healthcare professionals. For example, a previous study reported that early initiation of breastfeeding, early skin-to-skin contact, and rooming-in are associated with the continuation of EBF [22]. However, women commonly encounter challenges in breastfeeding practices during the first month, such as the baby’s failure to latch on, sore nipples, maternal feelings of insufficient milk supply, pain, and fatigue [23–25]. Milk supplementation may be used as an option to respond to these challenges. Previous studies conducted in Hong Kong, Vietnam, and the United States have reported that formula milk supplementation is associated with the early cessation of EBF [26–28] and failure to achieve the duration desired by women [29]. However, to our knowledge, no study has examined whether breastfeeding practices during hospitalisation are associated with subsequent practices after discharge in Japan.

Additionally, after discharge from the hospital until 5–6 months postpartum, there are significant changes in the mother’s health, daily life patterns, and the child’s growth. This may affect breastfeeding practices from month to month. Therefore, to better understand appropriate and timely breastfeeding support during and after hospitalisation, a study examining the association between breastfeeding status during hospitalisation and subsequent practice, considering changes in practice patterns over time, is required.

This study had two objectives. First, we aimed to examine the association between EBF during the first 7 d after birth (a proxy for the duration of hospitalisation) and EBF 24-h before the survey among children aged < 6 months and assess the strength of this association by comparing it with the association of EBF during the first day after birth. We also aimed to describe the changes in breastfeeding patterns from the first to the sixth month postpartum based on breastfeeding status during hospitalisation.

## Materials and methods

### Study design and population

This study was part of the Japan COVID-19 Society Internet Survey (JACSIS), a cross-sectional nationwide internet survey. JACSIS comprises three components: surveys of the general population, women, and male partners. The study participants for each survey were selected from the pooled panels of Rakuten Insight, Inc., an online research company with over 2.2 million panellists enrolled as of 2019 [30]. This study used data from a survey of women. The study population for the women’s survey was pregnant women who were expected to deliver by December 2021 and postpartum women who had a live singleton delivery between January 2019 and August 2021. A sample size estimation was not performed before the survey because the project aimed to examine multiple health and social topics associated with COVID-19. Therefore, we recruited the maximum number of eligible women from the pooled panel. During the recruitment process, a screening survey was conducted to identify 14,086 eligible women (11,661 postpartum and 2,425 pregnant women). Thereafter, they received email invitations to participate in the survey. Subsequently, 8,047 women (6,256 postpartum and 1,791 pregnant women) provided consent to participate in the survey between 28 July and 30 August 2021.

**Fig 1:**
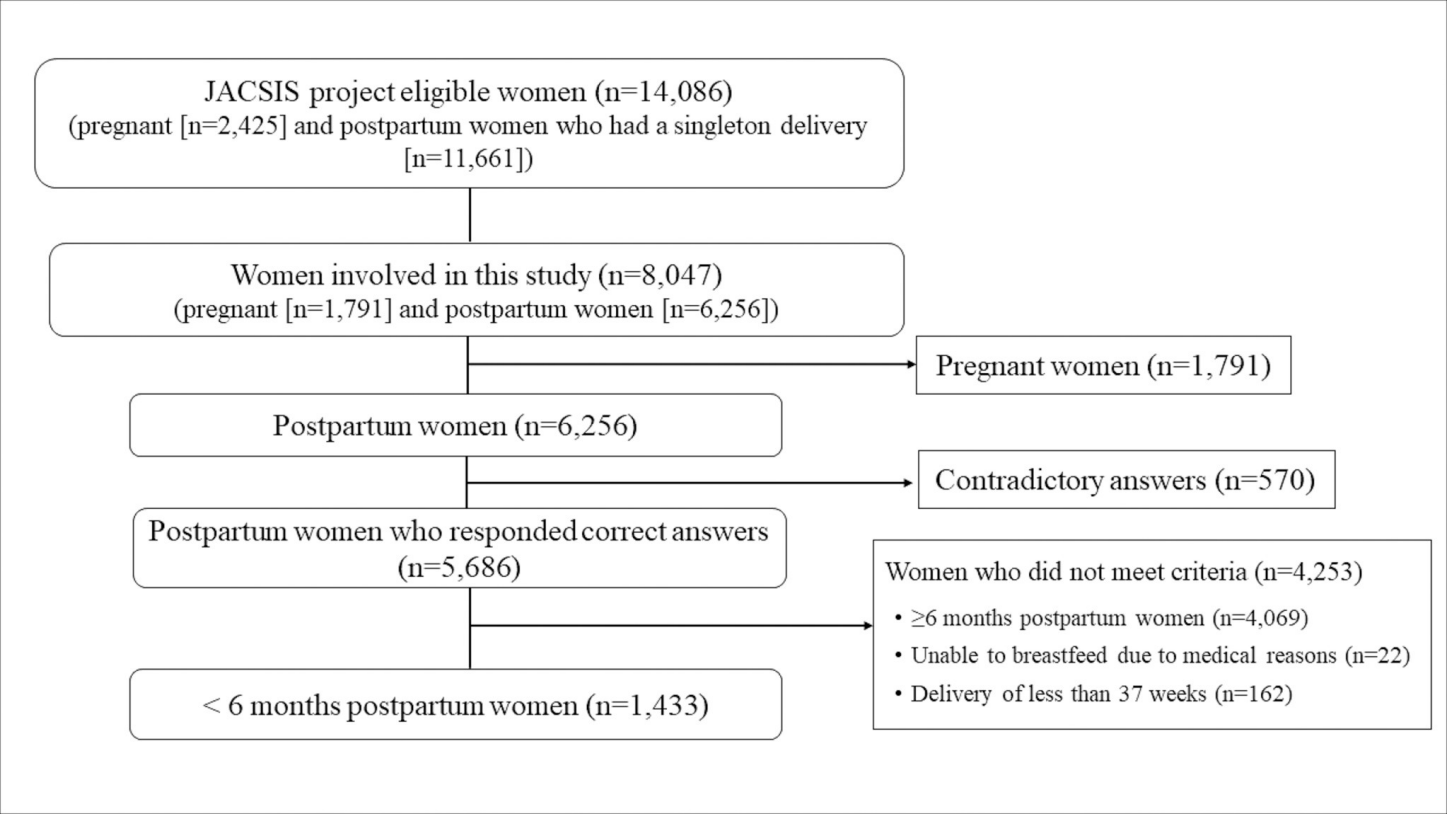
Flow diagram of the recruitment of study participants.

The following criteria were set for data analysis. The inclusion criterion was < 6 months postpartum. The exclusion criteria were as follows: 1) giving contradictory answers, 2) inability to breastfeed for medical reasons, and 3) preterm births before 37 weeks. This criterion was because women with preterm births were most likely to have difficulty practising EBF due to their children’s admission to the neonatal intensive care unit and their immaturity. Based on these criteria, of 6,256 postpartum women, 570 who gave contradictory answers and 4,253 who met the exclusion criteria of 2) or 3) were excluded. Overall, 1,433 postpartum women were included in the analysis (Fig 1). We evaluated whether the distribution of the participating women by prefecture of residence was almost equal to the distribution of births by prefecture in 2020 [31].

### Outcome variable: Exclusive breastfeeding of < 6 months measured using the 24-h recall method

Respondents were asked about their breastfeeding status 24 h before the survey. Their children were considered exclusively breastfed if they had been exclusively breastfed for the previous 24 h (referred to as 24-h recall EBF). This method follows the definition of EBF in the infant and young child feeding (IYCF) indicator published by WHO and UNICEF as “the proportion of infants aged 0–5 months who were exclusively breastfed in the preceding 24 h”, which is widely used in WHO and UNICEF data [32].

### Exposure variables: Exclusive breastfeeding during hospitalisation

We defined EBF during the first 7 d after birth (7-d EBF) as a proxy for EBF during hospitalisation based on the average duration of hospitalisation for singleton vaginal delivery in Japan [18, 19]. To compare the strength of the association of EBF during hospitalisation with EBF under 6 months, we used “EBF during the first day after birth” (first-day EBF) as another exposure variable. We used this variable as an alternative indicator of EBF for the first 2 d after birth, as recommended by the WHO and UNICEF, due to data availability constraints. In the survey questionnaire, the respondents were asked whether they gave breast milk and/or formula milk to their children during the first 24 h and during the first 7 d after childbirth. Women who responded with only breast milk but no formula milk during these periods were classified into the EBF group.

### Adjustment variables

The adjustment variables were selected based on previous similar studies [33, 34]. These variables were age (18–24, 25–29, 30–34, and 35–48 years), educational attainment (high school or lower, college/university/postgraduate), household income (low, lower middle, higher middle, high, declined to answer), parity (primipara, multipara), breastfeeding intention during pregnancy (only breast milk, others), mode of delivery (vaginal delivery, caesarean section), currently working indicating the working status at the time of the survey (yes, no), and the scores of the reception of support after discharge (score range 0–45). Reception of support after discharge was measured using the Breastfeeding Support Scale [35]. The scale comprises 11 items rated on a 5-point Likert scale ranging from 1 (strongly disagree) to 5 (strongly agree) [35]. We excluded two items, “Most health care providers (doctors, public health nurses, midwives, etc) support you in breastfeeding” and “Health care providers including doctors, public health nurses, or midwives tell me about the benefits of breastfeeding” because they were deemed to overlap with the support provided during hospitalisation [35]. Respondents who had never breastfed since birth (n = 46) were scored zero.

### Statistical analysis

First, we conducted a chi-square test and t-test to assess the distribution of participant characteristics based on their breastfeeding status during the first day and the first 7 d after birth, respectively.

Second, multivariate Poisson regression was performed to analyse the association of first-day EBF and 7-d EBF, with 24-h recall EBF before the survey. The strengths of the association were compared based on the prevalence ratio values. Poisson regression was employed because the prevalence of the outcome exceeded 10% [36].

**Fig 2:**
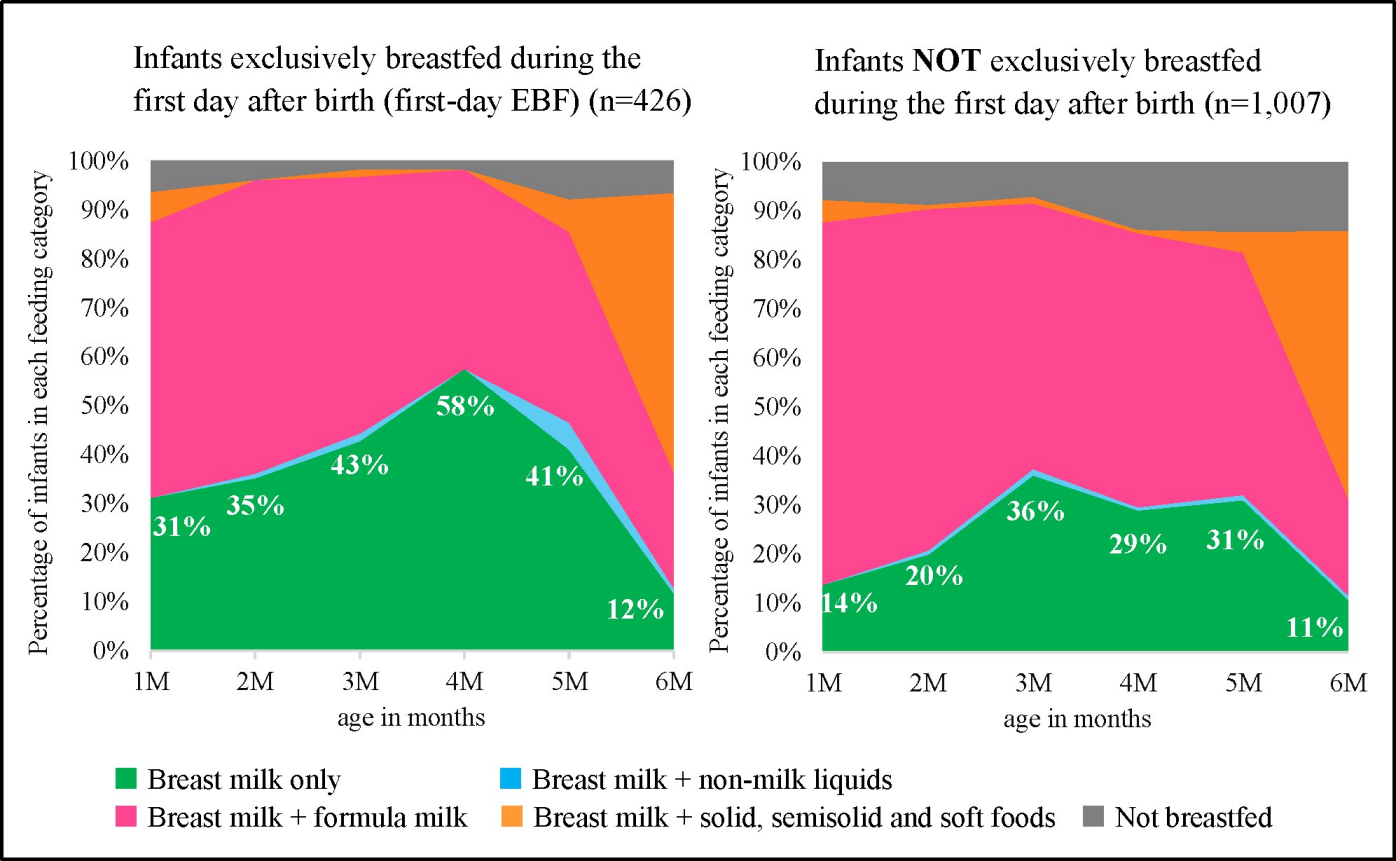
Area graph of breastfeeding status with and without exclusive breastfeeding during the first day after birth.

**Fig 3:**
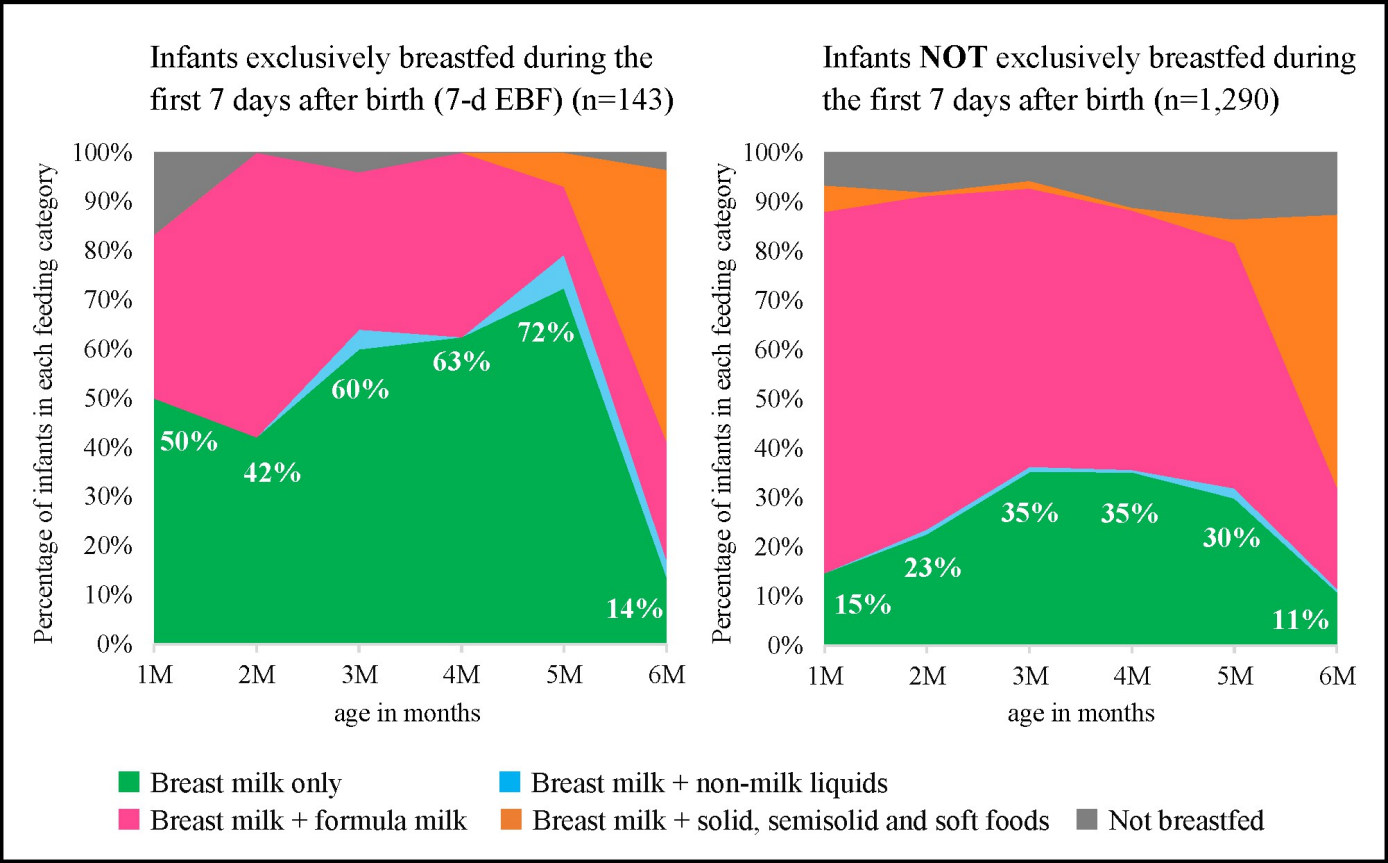
Area graph of breastfeeding status with and without exclusive breastfeeding during the first 7 d after birth.

Finally, we calculated the proportion of 24-h recall breastfeeding practices per age group under 6 months and described the results using area graphs (Figs 2 and 3). Fig 2 illustrates the breastfeeding status with and without exclusive breastfeeding during the first day after birth. Fig 3 shows the breastfeeding status with and without exclusive breastfeeding during the first 7 d after birth. The WHO and UNICEF recommend the area graph as part of the IYCF indicators for children aged 0–23 months, which can show the monthly trends of feeding practices [32]. WHO and UNICEF classify feeding practices into six patterns and plot the feeding status at 2-month intervals in children aged < 6 months; however, our study had to modify the six feeding patterns to five due to data limitations. These patterns were 1) breast milk only, 2) breast milk and non-milk liquids, 3) breast milk and formula milk, 4) breast milk and solid, semisolid, and soft foods, and 5) not breastfed. Additionally, we plotted feeding on a monthly basis to capture the Japanese patterns. We used statistical software, Stata version 15.0, for data analysis. Statistical significance was set at P < 0.05.

### Ethical considerations

The procedures used in this study complied with the ethical guidelines for medical and health research involving human participants, enforced by the Ministry of Health, Labor, and Welfare, Japan. We obtained an electrical informed consent form before commencing the survey, with clear information that they retained the option to withdraw from the study at any point. Data were collected anonymously from participants with strict confidentiality to maintain their privacy. Participants received credit points (“Epoints”) upon completion of the survey as a token of appreciation for their involvement in the study. This study was approved by the Bioethics Review Committee of Osaka International Cancer Institute, Japan (20084).

## Results

Overall, 1,433 postpartum women were included in the analysis. Table 1 shows the basic information of the participating women in the two groups: 426 women (29.7%) exclusively breastfed during the first day after birth, and 143 participants (10.0%) exclusively breastfed during the first 7 d after birth. The prevalence of the first-day EBF and the 7-d EBF groups differed significantly based on parity, breastfeeding intention during pregnancy, and mode of delivery. Notably, multiparous women, those who intended to provide only breast milk, and women who delivered vaginally were more likely to have breastfed exclusively during the first day and 7 d after birth. The prevalence of the 7-d EBF group was significantly different according to household income level, unlike that of the first-day EBF group.

**Table 1:** Basic information of the study participants (N = 1,433).

	Exclusively breastfed during the first day (first-day EBF)				Exclusively breastfed during the first 7 days (7-d EBF)			
	Yes		No		Yes		No	
	N	(%)	N	(%)	N	(%)	N	(%)
**Total** *	426	(29.7)	1,007	(70.3)	143	(10.0)	1,290	(90.0)
**Age**								
18–24	47	(69.1)	21	(30.9)	9	(13.2)	59	(86.8)
25–29	286	(66.1)	147	(33.9)	42	(9.7)	391	(90.3)
30–34	415	(73.8)	147	(26.2)	45	(8.0)	517	(92.0)
35–48	259	(70.0)	111	(30.0)	47	(12.7)	323	(87.3)
**Educational attainment**								
High school or lower	91	(32.3)	191	(67.7)	36	(12.8)	246	(87.2)
College/university/post-graduate	335	(29.1)	816	(70.9)	107	(9.3)	1,044	(90.7)
**Marital status**								
Not married	2	(16.7)	10	(83.3)	0	(0.0)	12	(100.0)
Married (Cohabitation)	416	(29.8)	979	(70.2)	141	(10.1)	1,254	(89.9)
Married (No cohabitation)	4	(23.5)	13	(76.5)	1	(5.9)	16	(94.1)
Divorced	4	(44.4)	5	(55.6)	1	(11.1)	8	(88.9)
**Household income**								
Low	95	(36.7)	164	(63.3)	39	(15.1)	220	(84.9)
Lower middle	78	(26.8)	213	(73.2)	25	(8.6)	266	(91.4)
Higher middle	97	(28.4)	244	(71.6)	24	(7.0)	317	(93.0)
High	99	(29.4)	238	(70.6)	34	(10.1)	303	(89.9)
Declined to answer	57	(27.8)	148	(72.2)	21	(10.2)	184	(89.8)
**Parity**								
Primipara	189	(26.1)	536	(73.9)	55	(7.6)	670	(92.4)
Multipara	237	(33.5)	471	(66.5)	88	(12.4)	620	(87.6)
**Breastfeeding intention during pregnancy**								
Only breast milk	234	(33.3)	469	(66.7)	92	(13.1)	611	(86.9)
Others	192	(26.3)	538	(73.7)	51	(7.0)	679	(93.0)
**Mode of delivery**								
Vaginal delivery	376	(32.0)	799	(68.0)	127	(10.8)	1,048	(89.2)
Caesarean section	50	(19.4)	208	(80.6)	16	(6.2)	242	(93.8)
**Currently working**								
Yes	18	(32.7)	37	(67.3)	8	(14.5)	47	(85.5)
No	408	(29.6)	970	(70.4)	135	(9.8)	1,243	(90.2)
**Support after discharge** **	32.0	(9.9)	31.7	(10.0)	30.0	(11.6)	32.0	(9.8)

* The percentages presented in this line indicate the proportion of those who answered ‘Yes’ or ‘No’ to the total number of participants.

**Support after discharge was a continuous variable presented as means and standard deviations.

Table 2 shows the associations of first-day EBF and 7-d EBF with 24-h recall EBF of < 6 months. First-day EBF (prevalence ratio [PR] 1.34; 95% confidence interval [CI], 1.14–1.58) and 7-d EBF groups (PR1.63; 95%CI, 1.35–1.96) showed significantly higher PRs of 24-h recall EBF of < 6 months, compared with non-EBF groups. Especially, the PR of the 7-d EBF group was higher than that of the first-day EBF group. Among the adjustment variables, multipara and intention to give only breast milk were also statistically significantly associated with 24-h recall EBF of < 6 months in both groups.

**Table 2:** Association of breastfeeding during the first day and the first 7 d after birth with 24-h recall EBF before the survey.

	Exclusive breastfeeding under 6 months of age (24‐h recall EBF)					
	PR	(95%CI)	P-value	PR	(95%CI)	P-value
**Exclusively breastfed during the first day (first-day EBF)**						
Yes	1.34	(1.14–1.58)	0.001	/	/	/
No	ref			/	/	/
**Exclusively breastfed during the first 7days (7-d EBF)**						
Yes	/	/	/	1.63	(1.35–1.96)	<0.001
No	/	/	/	ref		
**Age**						
18–24	ref			ref		
25–29	1.11	(0.71–1.74)	0.649	1.12	(0.71–1.75)	0.627
30–34	1.06	(0.68–1.67)	0.788	1.06	(0.67–1.66)	0.813
35–48	0.96	(0.60–1.53)	0.860	0.94	(0.59–1.50)	0.797
**Educational attainment**						
High school or lower	ref			ref		
College/university/post-graduate	1.04	(0.85–1.28)	0.705	1.05	(0.86–1.29)	0.620
**Household income**						
Low	ref			ref		
Lower middle	0.97	(0.76–1.24)	0.784	0.97	(0.76–1.24)	0.831
Higher middle	0.99	(0.78–1.27)	0.939	1.00	(0.79–1.27)	0.993
High	1.07	(0.84–1.36)	0.597	1.07	(0.84–1.36)	0.604
Declined to answer	0.93	(0.69–1.25)	0.624	0.93	(0.69–1.24)	0.621
**Parity**						
Primipara	ref			ref		
Multipara	1.57	(1.30–1.89)	<0.001	1.57	(1.30–1.89)	<0.001
**Breastfeeding intention during pregnancy**						
Others	ref			ref		
Only breast milk	2.69	(2.19–3.31)	<0.001	2.65	(2.16–3.26)	<0.001
**Mode of delivery**						
Vaginal delivery	ref			ref		
Caesarean section	1.05	(0.83–1.31)	0.701	1.04	(0.83–1.30)	0.755
**Currently working**						
Yes	ref			ref		
No	1.47	(0.81–2.67)	0.203	1.51	(0.83–2.78)	0.180
**Support after discharge** *	1.01	(0.98–1.03)	0.517	1.01	(0.99–1.04)	0.333

* These variables are measured as continuous data.

The area graphs depict the monthly proportions of each feeding pattern and show the difference in the area size between the EBF and non-EBF groups for the first day (Fig 2) and 7 d (Fig 3), respectively. Overall, the two figures highlight that the proportion of 24-h recall EBF increased from 1 to 4 months for the first-day EBF and 7-d EBF groups (left side of the Figs), which made the green area larger than their counterparts (right side of the Figs). The contrast between the EBF and non-EBF groups was more pronounced in Fig 3 than in Fig 2.

As shown in Fig 2, among infants exclusively breastfed during the first day, 24-h recall EBF rates began at 31% at 1 month of age and gradually increased to a peak of 58% at 4 months. Conversely, infants who were not exclusively breastfed on the first day showed consistently low 24-h recall EBF rates throughout the study period. Also, Fig 3 shows that the 24-h recall EBF rate for infants exclusively breastfed during the first 7 d was 50% at 1 month of age and increased steadily to 72% at 5 months. In contrast, for those who were not exclusively breastfed during the first 7 d, the 24-h recall EBF rate started at 15% at 1 month, slowly increased to 35% at 3 months, and then plateaued.

## Discussion

This cross-sectional study of 1,433 women within 6 months postpartum found that EBF rates on the first day and the first 7 d after birth were 29.7% and 10.0%, respectively. Moreover, the 7-d EBF was more strongly associated with the 24-h recall EBF of < 6 months than the first-day EBF. These findings were clearly visualised using area graphs. The area graph shows that the proportion of EBF was low in the first month of life and gradually increased over time. To our knowledge, this is the first study to show, using area graphs, an association between EBF during hospitalisation and subsequent practice until 6 months postpartum, considering the change in breastfeeding status over time [37].

Interestingly, the 7-d EBF, a proxy for the hospitalisation period in Japan [18, 19], was strongly associated with subsequent EBF practice. However, the percentage of the 7-d EBF was only 10%. Even among women who intended to breastfeed exclusively during pregnancy, the rate was only 13%. A previous study of 61 BFHs in Japan reported that approximately 72% of infants were exclusively or predominantly breastfed during hospitalisation [18], which is much higher than our results. However, we did not assess whether milk formula was used for medical indications. Consequently, assuming that BFHs in the above study only used formula milk when there was a medical indication, approximately 28% of infants require formula milk for a medical indication. This implies that most infants in this study were supplemented with formula milk despite having no medical indications.

There may be several reasons for unnecessary milk supplementation. First, the lack of explicit guidance on breastfeeding support in Japan, despite the existence of the Breastfeeding and Weaning Support Guide [14], may lead to different standards for supplementation in health facilities. This may allow routine supplementation and easy selection of formula milk without requiring medical assessment. Therefore, EBF during hospitalisation can be increased if healthcare professionals strengthen breastfeeding support for women who do not meet medical indications, considering their physical condition and intention to feed after childbirth. Second, there is a possible gap in the breastfeeding support provided by healthcare professionals, especially in mixed maternity wards where maternity patients and others stay together. Owing to the decrease in birth rates, the number of mixed maternity wards has increased in Japan, reaching 80.6% in 2013 [38]. A study in Japan involving 52 facilities in 2019 revealed that midwives and nurses offered breastfeeding support in 46 facilities (88.5%); however, 40% of these facilities lacked breastfeeding education, especially for nurses in mixed maternity wards [39], suggesting a potential gap in breastfeeding support. Therefore, the gap in breastfeeding support within health facilities can be mitigated by strengthening the knowledge and skills of healthcare professionals, especially nurses.

The area graphs show that EBF during hospitalisation influenced its continuation in subsequent periods. Interestingly, a unique feeding pattern was observed on the area graphs. The proportion of EBF was the lowest in the first month of age and gradually increased each month until the weaning period. This pattern contradicted those reported in other countries, predominantly LMICs, which illustrated the highest rate of EBF in the first months that decreased from month to month [37, 40, 41]. This unique situation in Japan implies that some mothers experience a transition from non-EBF to EBF status after discharge. This transition may be facilitated by women’s efforts stemming from their intention to breastfeed exclusively, as highlighted in the results section, where this intention during pregnancy was significantly associated with 24-h recall EBF under 6 months of age. Moreover, women’s daily efforts, increased breastfeeding experiences, and infant growth may also facilitate this transition. Additionally, breastfeeding support and continuation of care by healthcare professionals or peer supporters, even after discharge, are crucial to facilitate EBF for < 6 months [42]. In Japan, most postpartum women and newborns have routine check-ups at 2 weeks and 1 month after delivery at the health facilities where the women delivered their babies [43]. If they do not have any problems, there is no further check-up until a home visit within 4 months [44] and a health check-up at 3–6 months organised by the municipalities [45, 46]. In addition to routine check-ups, various optional breastfeeding support mechanisms are available in Japan. For instance, since 2021, municipalities have been obligated to make efforts to provide “Care services after childbirth”, including overnight stays, day services, and outreach programs [47, 48]. Breastfeeding counselling is offered at hospitals, maternity homes, and even during home visits. Although their availability varies depending on the area and offered services, and user fees are not standardised [48, 49], these optional breastfeeding support mechanisms may contribute to increasing the EBF rate under 6 months of age. However, this study did not thoroughly investigate the reasons for the transition to exclusive breastfeeding after discharge among those who were not exclusively breastfeeding during hospitalisation. Therefore, further research is warranted.

Additionally, the absence of legal measures for formula milk marketing in Japan may easily allow formula milk marketing even at health facilities [50–53], such as the free offering of formula milk samples to women at discharge. This may influence the ease of choosing formula milk when postpartum women perceive insufficient milk production. Consequently, formula milk supplementation due to perceived insufficient milk production may reduce the breastfeeding frequency of the mother, breast milk production and cessation of breastfeeding, even if they wish to breastfeed exclusively [54, 55]. Therefore, strengthening the continuum of breastfeeding support, such as providing breastfeeding guidance and information on accessible services for mothers both in antenatal and postpartum periods, would mitigate breastfeeding difficulties, promote and sustain breastfeeding practices [56].

The postpartum period in this study was amid the COVID-19 pandemic, implying that lack of support due to the COVID-19 pandemic affected the results of this study, as other studies in HICs reported that a reduction of breastfeeding support by healthcare professionals and peers negatively affected breastfeeding practices [57–59]. Our study did not find significant associations between receiving support after discharge and EBF for < 6 months. This may have been affected by other factors that were not assessed in this study. For example, the avoidance of home visits by certain postpartum women was caused by fear of infection, as reported by the Ministry of Internal Affairs and Communications [60]. Currently, the restrictions imposed by the COVID-19 pandemic have been relaxed, and life is returning to the pre-pandemic situation. Therefore, further research is required to assess breastfeeding status in the post-pandemic period and its influencing factors.

### Limitations

This study has some limitations. First, this study measured EBF of < 6 months using the 24-h recall method. Notably, several studies have revealed that the prevalence of EBF in infants aged < 6 months is overestimated when assessed using a single 24-h recall method [61, 62]. However, this method can minimise recall bias and is widely used in the standard IYCF indicators of the WHO and UNICEF. Second, 9.1% of the women were excluded because of contradictory answers before the analysis. Selection bias in the study participants may have affected the results. Third, it is undeniable that variations in women’s physical nature of breast milk production and critical health conditions after childbirth may impact breastfeeding status during hospitalisation. Therefore, EBF during hospitalisation may not be solely influenced by healthcare professional support. Fourth, the sample size may not be large enough to provide robust results for the proportion of 24-h recall breastfeeding practices (Figs 2 and 3), because it was disaggregated by the monthly age of children. Finally, there was no information on whether the children received formula milk supplementation for medical reasons, among other things. Further research is required to determine the reasons for formula milk supplementation and the criteria for supplementation in health facilities.

## Conclusions

EBF during hospitalisation is strongly associated with continued EBF practice for < 6 months. However, approximately 90% of infants were supplemented with formula milk rather than with breast milk at health facilities during this period. Therefore, improving breastfeeding support in health facilities by mitigating the gap in knowledge and skills among healthcare professionals and offering continued breastfeeding support during and after hospitalisation may contribute to increasing EBF for < 6 months and more mothers and infants will benefit from breastfeeding.
